# Administration of taurolidine-citrate lock solution for prevention of central venous catheter infection in adult neutropenic haematological patients: a randomised, double-blinded, placebo-controlled trial (TAURCAT)

**DOI:** 10.1186/s13063-018-2647-y

**Published:** 2018-05-02

**Authors:** C. Gudiol, S. Nicolae, C. Royo-Cebrecos, M. Aguilar-Guisado, I. Montero, C. Martín-Gandul, M. Perayre, D. Berbel, M. Encuentra, M. Arnan, J. M. Cisneros-Herreros, J. Carratalà

**Affiliations:** 1 Infectious Diseases Department, Bellvitge University Hospital, IDIBELL, Feixa Llarga SN, 08907 L’Hospitalet de Llobregat, Barcelona Spain; 2 Department of Infectious Diseases, Microbiology, and Preventive Medicine, University Hospital Virgen del Rocío-Institute of Biomedicine of Seville, Seville, Spain; 3 Department of Haematology, University Hospital Virgen del Rocío-Institute of Biomedicine of Seville, Seville, Spain; 4 Pharmacy Department, Clinical Trial Unit, Institut Català d’Oncologia, IDIBELL, L’Hospitalet de Llobregat, Barcelona Spain; 5 Microbiology Department, Bellvitge University Hospital, IDIBELL, L’Hospitalet de Llobregat, Barcelona Spain; 6 Biostatistics. Clinical Research Unit, Institut Català d’Oncologia, IDIBELL, L’Hospitalet de Llobregat, Barcelona Spain; 7 Haematology Department, Institut Català d’Oncologia, IDIBELL, L’Hospitalet de Llobregat, Barcelona Spain; 8 University of Barcelona, Barcelona, Spain; 9 REIPI (Spanish Network for Research in Infectious Disease), Instituto de Salud Carlos III, Madrid, Spain

**Keywords:** Catheter-related bloodstream infection, Central venous catheter infection, Antibiotic lock technique, Lock solution, Taurolidine-citrate, Haematological patients, Neutropenia

## Abstract

**Background:**

Catheter-related bloodstream infection (CRBSI) is one of the most frequent complications in patients with cancer who have central venous catheters (CVCs) implanted and is associated with substantial morbidity and mortality. Taurolidine is a non-antibiotic agent with broad-spectrum antimicrobial activity, which has been used as a lock solution to prevent CRBSI in some settings. However, little is known about its usefulness in high-risk adult neutropenic patients with cancer. This prospective randomised clinical trial aims to test the hypothesis that taurolidine-citrate lock solution is more effective than placebo for preventing catheter infection in neutropenic haematological patients.

**Methods:**

This study is a prospective, multicentre, randomised, double-blinded, parallel, superiority, placebo-controlled trial. Patients with haematological cancer who are expected to develop prolonged neutropenia (> 7 days) and who have a non-tunnelled CVC implanted will be randomised to receive prophylactic taurolidine-citrate-heparin solution using a lock technique (study group) or heparin alone (placebo group). The primary endpoint will be bacterial colonisation of the CVC hubs. The secondary endpoints will be the incidence of CRBSI, CVC removal, adverse events, and 30-day case-fatality rate.

**Discussion:**

The lock technique is a preventive strategy that inhibits bacterial colonisation in the catheter hubs, which is the initial step of endoluminal catheter colonisation and the development of infection. Taurolidine is a nontoxic agent that does not develop antibiotic resistance because it acts as an antiseptic rather than an antibiotic. Taurolidine has shown controversial results in the few trials conducted in cancer patients. These studies have important limitations due to the lack of data on adult and/or high-risk neutropenic patients, the type of catheters studied (tunnelled or ports), and the lack of information regarding the intervention (e.g. dwelling of the solution, time, and periodicity of the lock technique). If our hypothesis is proven, the study could provide important solid evidence on the potential usefulness of this preventive procedure in a population at high risk of CRBSI, in whom this complication may significantly impair patient outcome.

**Trial registration:**

ISRCTN, ISRCTN47102251. Registered on 9 September 2015.

**Electronic supplementary material:**

The online version of this article (10.1186/s13063-018-2647-y) contains supplementary material, which is available to authorized users.

## Background

Catheter-related bloodstream infection (CRBSI) is one of the most frequent complications associated with the long-term use of catheters. Patients with cancer usually have central venous catheters (CVC) implanted for an indefinite period for the administration of chemotherapy and other products and medications and are at increased risk of presenting with this complication, especially during the neutropenic phase. CRBSI in patients with cancer is associated with substantial morbidity and mortality, with excess costs and prolonged length of hospital stay [1–3]. Importantly, CRBSI can delay the administration of chemotherapy, which may negatively influence patient outcomes [4].

Gram-positive organisms, mainly coagulase-negative staphylococci (CNS) and *Staphylococcus aureus*, are the leading cause of CRBSI in patients with cancer [1]. However, an increasing number of episodes of CRBSI due to Gram-negative bacilli and fungi have been recently documented in some institutions [5–7]. In addition, an alarming emergence of antimicrobial resistance is being observed among Gram-negative bacteria infecting high-risk patients with malignancies [8, 9]. This is of special concern due to the associated increased mortality of bloodstream infections (BSI) due to resistant organisms in neutropenic cancer patients [10–12].

The most frequent mechanism of infection with long-term, non-tunnelled CVC use is catheter hub contamination during catheter manipulation by health personnel, leading to endoluminal colonisation and infection [13, 14]. Bacterial colonisation of catheter lumens leads to the development of a microbial consortium associated with the catheter surface and embedded in an extracellular matrix, named biofilm, which hinders antibiotic penetration and bacterial eradication [15]. Catheter removal and treatment with systemic antibiotics is the ‘gold standard’ approach for CRBSI [14, 15]. However, in neutropenic cancer patients, the removal of their ‘highly needed’ catheter is not always feasible, even when the diagnosis of catheter infection is established [16]. Therefore, prevention of CRBSI in these high-risk patients is crucial.

The use of lock therapy with different solutions as a method of preventing luminal catheter colonisation is an effective strategy in reducing CRBSI [17]. Lock therapy involves instillation of a solution into the catheter lumen for a variable period of time, depending on the solution at hand [18]. Moreover, the use of lock therapy decreases the incidence of CRBIS in some studies involving patients with cancer [19]. However, significant differences in the definitions of CRBSI exist in the studies performed, as well as in the type of catheters, the solutions used, and the dwelling times. Additionally, almost all studies have been performed in paediatric populations, and the number of high-risk neutropenic patients included in the studies is very small. In fact, national guidelines do not currently support the prophylactic use of lock therapy in patients with cancer, while waiting for higher-quality scientific evidence.

Vancomycin, ethanol, and taurolidine have been the most frequently studied lock solutions in patients with cancer, with very variable results, according to the significant aforementioned differences in the methodology of the studies and the population involved [20–29]. Of note, there is great concern regarding the use of vancomycin as a lock solution, since there is a risk of developing antibiotic resistance. Furthermore, the adverse events observed with the use of ethanol discourage its use as a lock solution. Taurolidine is a non-toxic substance derived from the amino acid taurine, which has anti-adherent, immunomodulatory, antithrombotic, and antitumour properties [30]. Although it is not an antibiotic, it has a wide array of antimicrobial activity against a broad spectrum of microorganisms, including Gram-positive bacteria, Gram-negative bacteria, and fungi. Taurolidine-citrate was found to be effective in two [28, 29] of the three studies involving paediatric patients with cancer, reducing CRBSI rates compared with heparin solutions, but it did not offer any benefit when used in the single adult study performed in patients with non-haematological cancer who had totally implantable venous access ports implanted [31]. The scarce and heterogeneous data obtained from these studies do not draw any solid conclusion. Moreover, almost all reported studies involved paediatric patients, and the devices studied in all of these trials were tunnelled catheters or ports.

This prospective, multicentre, randomised, double-blinded, placebo-controlled trial aims to test the hypothesis that lock solution with taurolidine-citrate-heparin is more effective than placebo for preventing CVC catheter infection in high-risk neutropenic haematological patients.

### Objectives of the study

This study aims to determine whether taurolidine-citrate-heparin lock solution is more effective than heparin alone for the prevention of CVC infection in neutropenic haematological patients.

### Primary endpoint

Bacterial colonisation of the CVC hubs.

### Secondary endpoints

Incidence of CRBSI, CVC removal, adverse reactions, and 30-day case-fatality rate.

## Methods

### Study design

This study is a prospective, multicentre, randomised, double-blinded, parallel, superiority, placebo-controlled trial (1:1). Patients will be randomised after completion of chemotherapy. Patients who were expected to develop prolonged (> 7 days) neutropenia will be randomised to receive a prophylactic solution of taurolidine-citrate-heparin using the lock technique (study group) or a solution with heparin alone using the same technique (placebo group). The study will be conducted in accordance with the Standard Protocol Items: Recommendations for Interventional Trials (SPIRIT) recommendations [32], Additional file 1.

### Study population

This study includes adult patients with haematological malignancies admitted to the haematology wards who are receiving chemotherapy, who are expected to develop prolonged neutropenia (> 7 days), and who have a non-tunnelled CVC implanted.

### Setting

The study will be conducted in Institut Català d’Oncologia L’Hospitalet (ICO Hospitalet), a 100-bed capacity hospital for adult patients, a referral cancer centre for a population of one million people. University Hospital of Bellvitge (HUB), an 800-bed-capacity tertiary hospital for adult patients. Both hospitals are located in the same health complex and are part of the Institute of Biomedical Research of Bellvitge (IDIBELL). Hospital Universitario Virgen del Rocío (HUVR) is a 1700-bed capacity hospital that caters to half a million inhabitants in the province of Seville.

### Selection of cases and recruitment

Patients admitted in the haematology wards of the two participating centres receiving myeloablative chemotherapy and who are expected to develop prolonged neutropenia (> 7 days) will be followed up daily by the attending physicians and by a research nurse specifically commissioned to carry out this study. Screening swabs from the catheter hubs and the catheter insertion site will be collected previous to randomisation by the usual nursing personnel; then, the catheter samples will be sent to the microbiology laboratory in order to identify patients with positive cultures (exclusion criteria). Patients with negative screening cultures will be followed up daily by the research nurse in charge and/or the investigators of the study, to assess whether they meet all the inclusion criteria and none of the exclusion criteria. A blood test will be performed every 2–3 days according to the protocols of each centre to document the development of neutropenia. Patients with neutropenia who met the inclusion criteria will be asked to sign the informed consent. The research nurse in charge and/or the investigators of the study will explain the study to the potential participants and ask them to sign the informed consent.

### Inclusion criteria

1. Patients aged 18 years or older who are admitted to the hospital because of leukaemia, lymphoma or to undergo haematopoietic stem-cell transplantation (HSCT)
2. Carriers of multiple-lumen, non-tunnelled CVCs, including peripherally inserted central catheters (PICCs)
3. Patients who are expected to develop neutropenia (< 500 neutrophils/μL) lasting more than 7 days

A patient could be included twice if two independent periods of neutropenia occur with at least 1 month interval between them. This interval will be calculated from the final visit (30 days from the end of the intervention) to the new inclusion. In this cases, patients will be randomised again.

### Exclusion criteria

1. Patients with a (non-flexible) semi-rigid or tunnelled CVC or totally implantable venous access ports
2. Patients who have been found to have a positive culture of the hubs or the insertion site of the CVC based on the results of the baseline screening cultures
3. Patients in whom the CVC is indispensable for the use of continuous infusion medication
4. Patients receiving systemic antibiotic treatment ≥ 48 h prior to study entry
5. Patients with an active infection

### Randomisation

In this randomised, double-blinded study, neither the patient nor any of the health staff will know which product is assigned to each patient.

A unique list of coded numbers containing the randomised solutions will be generated in the Clinical Research Unit. The randomisation will be performed in each block and stratified. The stratum will contain three groups: patients with leukaemia, patients undergoing an autologous transplantation, and patients undergoing an allogeneic transplantation. The random number generator of SAS version 9.2 will generate the three lists. Once generated, the list will be kept in the pharmacy file, in the pharmacy department of ICO Hospitalet with restricted access to the blinded personnel involved in the trial. When a patient from any of the two participating centres meets the inclusion criteria and signs the informed consent, an email with the recruitment sheet of the patient will be sent to the pharmacy department of ICO Hospitalet, where the randomisation will take place, by the investigator from the pharmacy department in charge of the study. The assignment will be made sequentially, and each patient will be assigned a randomisation number.

The taurolidine-citrate-heparin and the heparin-placebo solutions will be prepared in each pharmacy department, and they will be dispensed in 2.5-mL syringes identified with the name of the drug, defined as heparin/taurolidine-citrate, to maintain the blinding. Citrate is an anticoagulant substance without antimicrobial activity, which accompanies taurolidine as an antiaggregant agent. Both lock solutions are colourless and odourless, which makes further masking unnecessary. The blind will be open before the statistical analysis, but only when the trial has concluded and the definitive data will be registered in the database. Only in cases with suspected serious adverse events will the blinding be open.

### Intervention

Once the randomisation has been performed, a syringe containing 2.5 mL of the assigned lock solution (taurolidine 2%-citrate 4%-heparin 500 UI/mL, or heparin 1% alone at a dose of 1000 UI/mL) will be injected, by usual haematology ward nurses, through each catheter lumen three times a week, approximately every 48-72 h. The solution will be allowed to dwell for approximately 2 h. In all cases, the solution will be aspirated with a syringe and discarded. Before the lock technique, cultures of the hubs and the CVC insertion site will be taken in order to assess the bacterial colonisation of these sites. The research nurse in charge of the study will supervise all the proceedings related with the intervention. Also, the nurse in charge will periodically provide a report to the principal investigator of the study, informing whether the interventions have been performed properly.

### Catheter management

Multilumen, non-tunnelled CVCs will be inserted into the vein by a specifically trained staff, under the standard precautions. At the time of the catheter placement, the insertion site will be disinfected with 2% chlorhexidine in an alcohol base and then covered with a transparent dressing, except in patients with allergy to this type of dressing, in whom sterile gauze will be used. Also, when there is any sign of inflammation at the insertion site, the dressing will be replaced by a chlorhexidine-impregnated dressing. The care of the CVCs will include the replacement of the dressings every 6–10 days, or before if necessary. Before any manipulation, the catheter hubs will be disinfected using an external clean with 70° alcohol. If the catheter is sealed, the hubs will be disinfected every 6–10 days. If any of the catheters lumens become sluggish or occluded, flushing with urokinase will be performed. Any change of these procedures will be recorded in the database. The manipulations of the CVCs will be carried out under maximum barrier measures.

### Follow-up criteria and participant timeline

Patients will continue to receive intervention until one of following events occurs:

1. Positivity of any of the cultures taken from the CVC hubs
2. Recovery of neutropenia: > 500 neutrophils/μL
3. Removal of the catheter for any cause
4. Episode of CRBSI
5. Need to use the CVC for the administration of a medication in continuous infusion that cannot be stopped for any reason
6. Discharge from the haematology ward for any reason, despite not having recovered from the neutropenia
7. Death

Once the patient concludes the study for any of the previous events, a 30-day follow-up will take place to assess possible complications until that date. The intervention will be stopped in patients in whom significant colonisation of a CVC hub is detected, but they will continue to be followed-up to check whether they develop CRBSI. Whenever the catheter is removed on suspicion of infection, the hubs and tip of the catheter will be sent for microbiological culture.

**Fig. 1:**
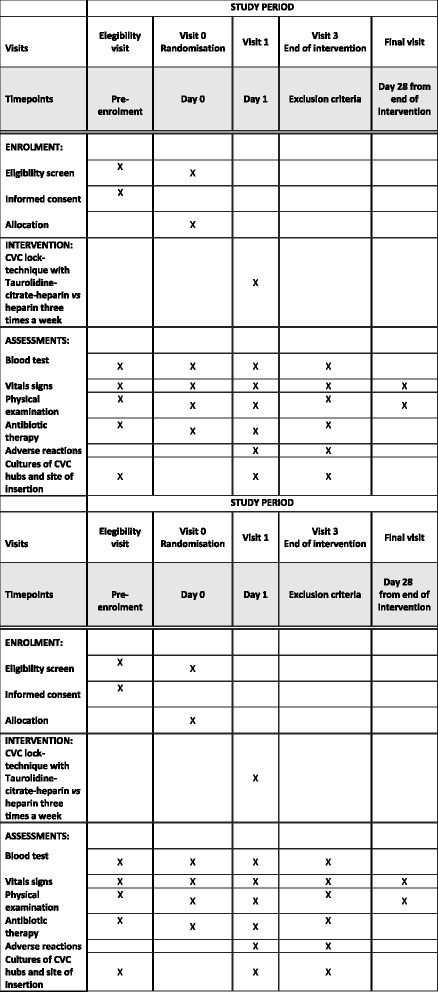
Participant timeline of the study

A patient will be considered to have discontinued the study when the patient withdraws the informed consent, dies, or is lost to follow-up. The trial will be considered finished when all the recruited participants have completed the 30-day follow-up period. Figure 1 shows the participant timeline of the study.

### Interruption criteria

1. Withdrawal of consent
2. Violation of masking
3. Violation of the inclusion/exclusion criteria
4. Serious adverse reaction

In accordance with the current revision of the Declaration of Helsinki and the applicable regulations, a patient has the right to withdraw from the study at any time and for any reason, without this causing any harm to the medical care by their physician. The withdrawal of full consent from a study means that the patient does not wish to continue participating in the study. Any patient withdrawing their consent will be removed from the treatment and/or the study observations immediately after the date the patient requests it.

### Data collection

The following data will be collected: sex, age, type of underlying disease, other comorbidities, date of hospital admission, HSCT, type and date of HSCT, immunosuppressive therapy, concomitant medication, duration of neutropenia, type of CVC, days from CVC insertion to inclusion in the study, use of prophylactic antibiotics or antifungal agents, systemic antibiotic therapy, number of catheter locks per episode, results of the cultures of the hubs and CVC insertion site, any episodes of infection, episodes of CRBSI, the need for CVC removal and reason, development of complications, adverse reactions, overall case-fatality rate, CRBSI attributable case-fatality rate, and date of discharge.

### Definitions

Significant colonisation of any catheter hub will be defined as the presence of any microbial growth in the qualitative culture of the swabs. CRBSI will be defined as the detection of growth of bacteria or fungi in at least one blood culture obtained from a peripheral vein, associated with clinical signs and symptoms of infection (e.g. fever, chills, and/or hypotension), and no apparent source for BSI other than the CVC. In the case of common skin-colonising microorganisms, such as CNS, two positive sets of blood cultures will be necessary to make the diagnosis of CRBSI. CRBSI attributed to luminal colonisation will be defined as the isolation of identical organisms from the catheter hub and from cultures of separate percutaneously drawn blood specimens; the organism’s identity will be proven by molecular typing for patients with BSI due to CNS. Overall, the 30-day case-fatality rate will be defined as death by any cause within the 30 days following the end of the intervention.

### Study outcomes and endpoint assessment

#### Primary endpoint

For the assessment of the primary endpoint (bacterial colonisation rate of the CVC hubs), swabs of the inner surfaces of the catheter hubs will be obtained by the nursing personnel before randomisation and three times weekly (approximately every 48-72 h) thereafter for culture, until the end of the intervention. All swabs are made of viscose, and only the swab of the catheter insertion site has a transport media (Stuart media). One swab is used for each lumen The cultures of the collected swabs will be processed in the microbiology department. The results of the cultures will be reviewed by the participating investigators and will be written down in the data collection form and in the specific database.

#### Secondary endpoints

The assessment of the secondary endpoints will be performed as follows:

1. Incidence of CRBSI: swabs of the insertion site and catheter hubs as well as blood cultures (at least one from a peripheral vein) will be obtained when CRBSI is suspected. In the case of catheter removal, two 5-cm segments, a proximal subcutaneous segment and the tip, will be sampled using the sterile technique for culture. The incidence of CRBSI and the CRBSI attributed to luminal colonisation will be calculated for each group. The differential time to positivity will not be used to diagnose CRBSI. The development of CRBSI will be assessed during intervention and up to 30 days after the last intervention. The rate of CRBSI will be presented as the number of episodes of CRBSI per 1000 admission days
2. CVC removal: whenever it is necessary to remove the CVC because of occlusion, thrombosis, dislodgement or suspicion and/or documentation of infection, the tip is going to be sent for culture to the microbiology department, and the reasons for removal will be annotated in the data collection form and the database by the participating investigators. The need for CVC removal will be assessed during intervention and up to 30 days after the last intervention
3. Adverse reactions: any adverse reaction potentially associated with the administration of the lock solution will be annotated in the data collection form and the database by the participating investigators. Adverse events will be identified and classified according to the severity and the potential relationship with the lock solution. They will be assessed during the intervention
4. Overall (30-day) case-fatality rate: all patients included in the study will be followed up for a total of 30 days after the end of the intervention by the participating investigators. Death by any cause will be annotated in the data collection form and the database

The research nurse in charge of the study will visit the patients daily in order to collect all the data related to the study. Also, the nurse will assess the potential development of any adverse event, and will collect all the information in the database. Adverse events will be classified according to their severity and causality. Only the serious adverse events, and those considered to be related to the use of the product of the study will be reported to the study promoter and to the regulatory authorities.

### Sample size

According to our previous experience [26], we estimated that around 20% of patients in the control group will present positive cultures of the catheter hubs. A reduction of this figure to 5% in the taurolidine-citrate-heparin group would be clinically relevant. To detect a 15% difference between groups with a power of 80%, it would be necessary to include 135 patients in the study. Considering the possibility of a loss of 10% of cases, the final sample should be 150 patients.

### Statistical analysis

The analysis of the data will be carried out during the last 6 months of the project when all fieldwork has been completed. The database will then be analysed using the stepwise logistic regression model of the R software (R v. 3.2.5). The analyses will be made by intention-to-treat (all patients randomised who have a primary endpoint available). To handle missing data multiple imputation will be done under the missing at random assumption.

For the analysis of the primary endpoint, the frequency of colonisation of the CVC hubs in both treatment groups will be compared. For the analysis of the secondary endpoints, the frequency of CRBSI will be compared between the two groups, as well as the need for CVC removal for any cause and overall case-fatality rate (30 days). Exploratory subgroup analyses will be conducted by underlying haematological condition (leukaemia, autologous HSCT, and allogeneic HSCT) and by centre. Due to the fact that a patient could have been included twice, the potential impact of independence violation will be assessed repeating the main analysis using patient as a cluster.

The statistical analysis will be carried out using a Student’s *t* test and a chi-square test. A multivariate analysis will be performed to verify the absence of confusion by other variables. In addition, free time of colonisation of the catheter and BSI will be analysed using Kaplan-Meier curves, and they will be compared between both groups by means of the log-rank test (Mantel-Cox test). A value of *p* = 0.05 will be considered statistically significant.

### Microbiology studies

Samples from the catheter hubs and insertion site will be obtained using sterile swabs and plated in blood agar plates. A qualitative culture of these samples will be performed, and they will be considered positive when the growth of a microorganism is detected. After catheter removal, the terminal portion (5 cm) of the catheter tip will be cultured using the roll-plate technique (Maki’s technique) [33]. A bacterial count > 15 CFU will be considered significant. Blood cultures will be processed with the usual system in the microbiology laboratories of the participating centres (BACTEC FX, Becton Dickinson, BD, Madrid, Spain). The antibiotic susceptibility will be tested by disc diffusion and microdilution methods following the European Society of Clinical Microbiology and Infectious Diseases (EUCAST) recommendations and breakpoints.

In the case of CRBSI due to coagulase-negative staphylococci (CNS), if the same species are isolated in any of the catheter hubs or tip and in the blood cultures, the strains will be preserved frozen for further analysis. CNS isolates from blood and catheter samples will be sent to the microbiology laboratory of HUB. Species identification will be confirmed using MALDI-TOF (MALDI Biotyper, Bruker Daltonics, Germany). Finally, isolates will be studied by pulsed-field gel electrophoresis after restriction with SmaI in order to determine the intrapatient genetic relationship following the criteria described by Tenover [34].

### Monitoring

In compliance with the standards of Good Clinical Practice, the promoter will carry out the monitoring tasks of the study following the approved monitoring plan. Among others, the tasks of monitoring will include the assessment of the correct application of the inclusion and exclusion criteria, the assessment of the quality of the data collected, the maintenance of the blind, the development and reporting of any adverse events, and the maintenance of the confidentiality of the patients. The need for a Data Monitoring Committee (DMC) was waived by the Ethic Committee because of the low impact on the safety of the patients in the study. Also, no interim analyses were planned.

### Publication plan

Results will be reported at conferences and in peer-reviewed publications. The first publication will be based on data from the two participating centres and will be analysed as stipulated in the protocol with the statisticians’ supervision. Any formal presentation or publication of data collected from this study will be considered as a joint publication by the participating investigators and will follow the recommendations of the International Committee of Medical Journal Editors.

## Discussion

This large prospective, multicentre, randomised, double-blinded, placebo-controlled trial aims to test the hypothesis that lock solution with taurolidine-citrate-heparin is more effective than placebo for preventing CVC catheter infection in high-risk neutropenic haematological patients and, consequently, CRBSI.

Various strategies to reduce the incidence of CRBSI have been evaluated, including the use of antibiotic-impregnated catheters, strict hygienic measures, eradication of *S. aureus* nasal carriage, and application of prophylactic antibiotic ointment on the insertion site [35]. However, catheter infection still occurs, especially with long-term CVC use. Given the integral role of long-term CVC use in the health care system, administering lock solutions to patients with these types of catheters remains an important option for the prevention and adjunctive treatment of CRBSI. The lock technique is a preventive strategy that inhibits bacterial colonisation in the catheter hubs, which is the initial step of endoluminal catheter colonisation and the development of infection [13, 14].

A wide variety of antibiotic lock solutions have been evaluated for clinical use, with the largest body of data available for vancomycin and gentamicin [17]. In this regard, in a meta-analysis, vancomycin-containing lock or flush solutions had shown to reduce CRBSIs and prolonged catheter survival in patients receiving haemodialysis [36].

Patients with cancer who have a CVC implanted are at special risk of developing CRBSI. Infection rates of 2.66 per 1000 line-days have been found in adult haematology/oncology and bone marrow transplant units [37]. Recently, lock solution technique as a preventive strategy for CRBSI in patients with cancer has been systematically reviewed [19]. The great majority of studies were performed in paediatric patients (10 studies), and only three were carried out in the adult population. Vancomycin was the most frequently studied solution because the great majority of infections are caused by Gram-positive bacteria. The results of vancomycin studies were variable; some studies showed a reduction of CRBSI in paediatric patients with cancer [23, 25], while others failed to do so [24]. Moreover, Carratalà et al. [26] demonstrated a significant reduction of CRBSI in neutropenic haematological patients using vancomycin as lock solution in the single study carried out in adults. Nevertheless, the widespread use of prophylactic antibiotics as catheter lock solutions may contribute to the development of antibiotic-selective pressure and resistance. In addition, the emergence of Gram-negative bacteria as an important cause of CRBSI raises the doubt about the current utility of vancomycin. Furthermore, three studies investigating ethanol as a lock solution in paediatric and adult patients with cancer showed discordant results [20–22]. Moreover, relatively frequent adverse effects have been reported with the use of ethanol-based solutions, which limits its use for this purpose [21, 22].

Taurolidine is a non-toxic agent with antimicrobial activity against Gram-positive bacteria, Gram-negative bacteria, and fungi. It reduces biofilm formation and does not develop antibiotic resistance because it acts as an antiseptic rather than an antibiotic [30]. Interestingly, due to its broad-spectrum antibacterial activity, taurolidine-citrate used as a lock solution not only reduced the risk of CRBSI but also the risk of Gram-negative infections according to the results of a recent meta-analysis conducted in the general population without cancer [18]. In the aforementioned systematic review in patients with cancer by Norris et al. [19], taurolidine showed beneficial results compared with placebo in two of the three studies involving paediatric patients with cancer [28, 29]. On the contrary, a third study of paediatric patients with totally implantable venous access ports showed no beneficial effect [27]. Likewise, in a recent prospective, randomised, phase IV trial, taurolidine-citrate did not show a significant reduction in the risk of infection when used in adult patients with non-haematological cancer [31]. These studies have important limitations due to the lack of data on adult and/or high-risk neutropenic patients, the type of catheters studied (tunnelled or ports), and the lack of information regarding the intervention (e.g. dwelling of the solution, time, and periodicity of the lock technique).

The use of taurolidine-citrate as a lock solution has been associated with mild and self-limited adverse events when used as flushing, with an unpleasant taste in the mouth as the most common side effect.

In this study, we aim to assess the efficacy of taurolidine-citrate as a catheter lock solution in preventing CVC bacterial colonisation and, consequently, CRBSI in neutropenic haematological patients. If our hypothesis is proven, the study could provide important solid evidence on the potential usefulness of this preventive procedure in a population at high risk of CRBSI, in whom this complication may significantly impair patient outcome.

## Trial status

Recruitment of patients started on 1 January 2013, and it will be completed by 30 June 2018. Protocol version number 5.0. Date: 27 July 2016.

## Additional file

Additional file 1: SPIRIT 2013 Checklist: recommended items to address in a clinical trial protocol and related documents*. (DOCX 36 kb)

## Abbreviations

BSI: Bloodstream infection
CNS: Coagulase-negative staphylococci
CRBSI: Catheter-related bloodstream infection
CVC: Central venous catheter
EUCAST: European Society of Clinical Microbiology and Infectious Diseases
HSCT: Haematopoietic stem-cell transplantation
HUB: University Hospital of Bellvitge
HUVR: Hospital Universitario Virgen del Rocío
ICO Hospitalet: Institut Català d’Oncologia L’Hospitalet
IDIBELL: Institute of Biomedical Research of Bellvitge
PICC: Peripherally inserted central catheter
